# Effects of Periodontal Therapy on Cardiovascular Risk Biomarkers: A Systematic Review

**DOI:** 10.3290/j.ohpd.c_2173

**Published:** 2025-08-22

**Authors:** Camille Bechina, Ange Désiré Pockpa, Gilles Amador Del Valle, Assem Soueidan, Guillaume Lamirault, Xavier Struillou

**Affiliations:** a Camille Bechina Assistant, Department of Periodontology, Faculty of Dental Surgery, Nantes Université, Nantes. Idea, study design, data collection, data interpretation, wrote the manuscript.; b Ange Désiré Pockpa Assistant, Department of Periodontology, Faculty of Dental Surgery, University of Felix Hou-phouët Boigny, Abidjan, Ivory Coast. Contributed to the acquisition, analysis or interpretation of the data, read and approved the manuscript.; c Gilles Amador Del Valle Associate Professor, Department of Prevention, Epidemiology, Health Economics, Forensic-Odontology Law, UIC Odontology 11, Nantes, France. INSERM, Regenerative Medicine and Skeleton, RMeS, Nantes, France. Contributed to critical proofreading and discussion, read and approved the manuscript.; d Assem Soueidan Professor of Periodontology, CHU Nantes, Nantes Université, Periodontology Department, UIC Odontology 11, Nantes, France; Nantes Université, CHU Nantes, INSERM, Regenerative Medicine and Skeleton, RMeS, Nantes, France. Contributed to critical proofreading and discussion, read and approved the manuscript.; e Guillaume Lamirault Professor of Cardiology, Nantes Université, CHU Nantes, CNRS, INSERM, l’Institut du Thorax, F-44000 Nantes, France. Contributed to critical proofreading and discussion, read and approved the manuscript.; f Xavier Struillou Associate Professor of Periodontology, CHU Nantes, Nantes Université, Periodontology Department, UIC Odontology 11, Nantes, France; Nantes Université, CHU Nantes, INSERM, Regenerative Medicine and Skeleton, RMeS, Nantes, France. Contributed to the conception, acquisition, analysis or interpretation of the data, read and approved the manuscript.

**Keywords:** atherosclerosis, cardiovascular risk, periodontitis, peripheral arterial disease, therapy

## Abstract

**Purpose:**

Periodontal and cardiovascular diseases are prevalent chronic conditions sharing common pathogenic pathways involving bacterial translocation and systemic inflammation. This systematic review aimed to assess the impact of non-surgical periodontal therapy (NSPT) on cardiovascular risk biomarkers, including endothelial function, systemic inflammation and thrombosis markers, and lipid and glucose metabolism, in patients with or without comorbidities.

**Materials and Methods:**

A systematic search was conducted in PubMed, Cochrane Library, and Scopus databases, following preferred reporting items for systematic reviews and meta-analyses (PRISMA) guidelines and supplemented by manual searches. Eligible studies were published after 2010, written in English, and involved adult patients with moderate to severe periodontitis treated with NSPT. Risk of bias was assessed for all included studies.

**Results:**

Sixteen studies were included. NSPT was associated with a significant reduction in high-sensitivity C-reactive protein (hs-CRP) and proinflammatory cytokines (IL-6, TNF-α), as well as with decreased HbA1c levels, particularly in patients with type 2 diabetes. Improvements in endothelial function were observed, notably a reduction in endothelial microparticles (EMPs), although results across vascular parameters such as flow-mediated dilation (FMD) and PWV were heterogeneous. Effects on lipid profiles were inconsistent and generally modest.

**Conclusion:**

NSPT shows moderate to high clinical relevance by improving key cardiovascular biomarkers especially inflammation and glycaemic control with both healthy and comorbid patients. These findings support the integration of periodontal care into cardiovascular risk mana-gement strategies, though further research is needed to confirm effects on lipid metabolism and vascular function.

Periodontal diseases and cardiovascular diseases are among the most common multifactorial chronic diseases affecting people worldwide. Numerous studies have highlighted the high prevalence of these diseases, which are expected to increase worldwide in the coming years.^54,55^

Periodontal diseases are multifactorial infectious diseases characterised by inflammatory expression. They are characterised by clinical symptoms and signs that may include periodontal pocket formation, recessions, attachment loss, alveolar bone loss, variable tooth mobility associated with visible or invisible inflammation, and spontaneous or induced gingival bleeding. These factors can ultimately lead to tooth loss. Risk factors such as smoking, diabetes or systemic diseases can also exacerbate periodontal disease.^27,40^ According to the new classification (Chicago 2017), periodontitis is defined according to its severity, classified in stages from I to IV. Moderate periodontitis corresponds to stage II, characterised by clinical attachment loss of 3 to 4 mm and bone loss of between 15% and 30%. Severe periodontitis, on the other hand, corresponds to stages III or IV. It is manifested by a clinical loss of attachment of at least 5 mm and bone loss reaching the middle third or more of the root. Stage IV represents a more advanced form than stage III, with major tissue destruction, more serious functional and aesthetic consequences, and treatments requiring more complex management.^39^

According to the World Health Organization (WHO), severe periodontal diseases are estimated to affect approximately 19% of the global adult population. Trindade et al. reported that between 2011 and 2020, periodontitis in dentate adults was estimated to account for approximately 62%, and severe periodontitis accounted for 23.6%.^55^

In 2020, the WHO estimated that cardiovascular disease was responsible for almost 18 million deaths. Cardiovascular diseases are a group of disorders affecting the heart and blood vessels, including coronary heart disease, cerebrovascular disease, rheumatic heart disease and other conditions. The risk of cardiovascular disease is increased by socioenvironmental, metabolic and behavioural risk factors such as smoking, hypercholesterolemia, hypertension, diabetes, a sedentary lifestyle, abdominal obesity and psychosocial disorders.18,59 Cardiovascular diseases are often associated with atherosclerosis, an inflammatory disease that develops throughout an individual’s life and is characterised by phases of activity and rest.^32,42^

In recent years, numerous biochemical parameters have been evaluated as potential cardiovascular risk factors. These cardiovascular risk biomarkers include markers of inflammation, lipoproteins, haemostasis and oxidation.^4,61,62^

The link between periodontal diseases and systemic diseases has been established for many years.^13,14,37,44,63^ These connections are attributed to the inflammatory components of periodontal disease and the ability of periodontal inflammatory biomarkers to migrate into the general bloodstream. Periodontal pathogens can penetrate the endothelial and muscle cells of blood vessels. Numerous studies have also investigated the link between periodontal diseases and cardiovascular diseases, as well as the associated cardiovascular risk factors. The systemic inflammation associated with periodontitis, characterised by the activation of immune cells and the production of proinflammatory cytokines, promotes LDL oxidation and endothelial damage. These processes are key elements in the development of atherosclerotic plaque. Furthermore, the presence of periodontopathogen bacteria, such as Porphyromonas gingivalis, within atheromatous lesions exacerbates local inflammation and accelerates the progression of atherosclerosis.^5,45,47,58,60^

This systematic review aimed to study the impact of non-surgical periodontal therapy on cardiovascular risk biomarkers, such as endothelial function, markers of systemic inflammation and thrombosis and lipid and glucose metabolism, in patients with or without associated comorbidities.

## MATERIALS AND METHODS

The protocol for this review was registered in the Prospective International Registry of Systematic Reviews (PROSPERO) under ID CRD42024493295 and followed the 2020 preferred reporting items for systematic reviews and meta-analyses (PRISMA) guidelines.^25^

The PICOS structure was used to define the inclusion criteria and formulate the research question. Studies that met the following criteria were included:

Participants (P): Adult patients with moderate to severe chronic periodontitis, with or without associated comorbidities.
Interventions (I): Non-surgical periodontal therapy included supra- and subgingival scaling, root planning and oral hygiene instructions.
Comparison (C): Comparisons of the effects of periodontal therapy on cardiovascular risk biomarkers between the test group (which received periodontal therapy) and the control group (which did not receive periodontal therapy or receive limited dental prophylaxis).
Outcome (O): The main outcomes were the levels of cardiovascular risk biomarkers, including endothelial function, markers of systemic inflammation, and lipid and glucose metabolism. The additional outcomes concerning the periodontal treatment were levels of clinical attachment, probing pocket depths and bleeding on probing.
Study design (S): Only randomised controlled trials (RCTs) were selected.

### Search Strategy

The literature search for articles published from 2010 to 2024 was carried out using PubMed, the Cochrane Library and Scopus. A specific search equation was formulated for each database, using keywords and MeSH terms, as summarised in Table 1.

**Table 1:** Databases and search terms

	
Pubmed (filters applied: randomised control trial)	cardiovascular risk[Title/Abstract] OR Peripheral Arterial Disease[Title/Abstract] OR (Peripheral Arterial Disease[MeSH Terms] OR Vascular Stiffness[Title/Abstract] OR vascular stiffness[MeSH Terms] OR atherosclerosis[MeSH Terms] OR atherosclerosis[Title/Abstract] AND periodontal disease[MeSH Terms] OR periodontal diseases[MeSH Terms] OR chronic periodontitis[MeSH Terms] OR periodontitis[Title/Abstract] OR periodontal disease[Title/Abstract]
Cochrane library (all text)	cardiovascular risk OR Peripheral Arterial Disease OR Vascular Stiffness OR atherosclerosis AND periodontal disease OR chronic periodontitis OR periodontitis
Scopus (article title, abstract, keywords)	cardiovascular risk OR Peripheral Arterial Disease OR Vascular Stiffness OR atherosclerosis AND periodontal disease OR chronic periodontitis OR periodontitis


The method used was a systematic review of the literature. The research and article selection processes were carried out independently by two authors (CB and XS). The two reviewers manually searched for additional published papers that could meet the eligibility criteria of this study. The reviewers (CB and XS) independently performed a methodical analysis of all the study titles, abstracts, and full texts.

### Article Selection and Data Extraction

The titles and abstracts of the obtained articles were screened on the basis of the determined eligibility criteria. The full texts of the remaining studies were assessed by the same authors. The data of the included studies, when available, were extracted by both reviewers (CB and XS). A table was previously established to provide support for the collection of the following data: (1) Author, Year of Publication and Country; (2) study design; (3) number of patients (mean age ± SD); (4) follow-up; (5) study population; (6) intervention; (7) periodontal assessment; and (8) outcome measures.

### Quality Assessment

The risk of bias assessment was performed with Cochrane’s Collaboration tool for assessing the risk of bias in RCTs. Two authors (CB and XS) assessed and discussed the risk of bias in the included studies. The risk of bias level was determined to be low, unclear, or high according to the following criteria: (1) generation of the randomisation sequence (selection bias), (2) concealment of the allocation (reporting bias), (3) blinding of the investigators and the participants (confusion bias), (4) blinding of the evaluation of the results (performance bias), (5) management of missing data (attrition bias), (6) selection of the reporter, and (7) other types of bias.^52^

## RESULTS

### Study Selection

**Fig 1:**
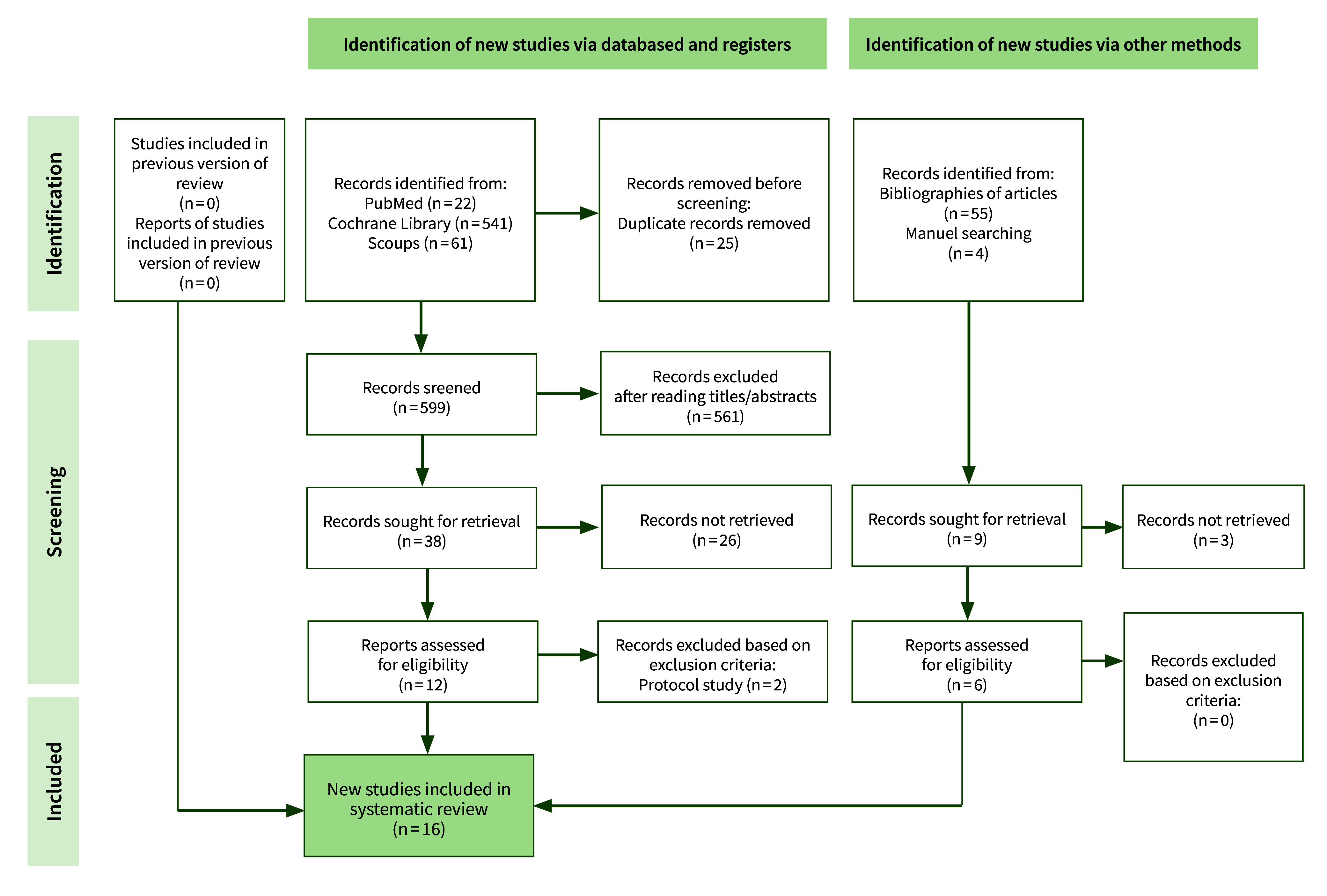
PRISMA flow diagram.^38^

The bibliographic search of all the sources identified 633 articles (624 from databases and 9 from other methods). Of these, 25 duplicate studies were removed via the reference manager EndNote. A total of 561 articles were excluded after the titles and/or abstracts were read, and 29 records were excluded since the reports were not retrieved. A total of 18 full-text records were read and analysed, and 2 more records were excluded because they were protocol studies, as shown in the PRISMA flowchart in Figure 1. Sixteen records met the inclusion criteria and were finally included in the systematic review.

### Description of the Included Studies

The characteristics of the sixteen articles included are presented in Table 2, with detailed information on the study design, population characteristics, duration of follow-up, interventions, periodontal assessment, and cardiovascular outcomes.

**Table 2:** Characteristics of included studies

Author,year of publication,country	Study design	No. of patients(mean age ± SD)	Follow-up	Study population	Intervention	Periodontal assessment	Outcome measures
Kolte et al, 2023 India^19^	RCT	CG = 30 TG = 30 The age range of 30–60 years.	Clinical follow-up at 3- (periodontal status only) and 6- months	Adults with controlled T2DM and Stage III periodontitis	CG: dental prophylaxis (supragingival plaque removal, oral hygiene instruction) TG: non-surgical periodontal therapy (supragingival plaque control, oral hygiene instruction, subgingival scaling and root)	PPD, CAL, BOP	IL-10, TNF-α, hs-CRP, Fasting blood glucose, post-meal blood glucose, HbA1c
Milanesi et al, 2022 Brazil^33^	RCT	CG = 79 TG = 79 Missing Data	Clinical follow-up at 3- and 6- months	MetS patients with periodontitis stage 3-4 grade B/C	CG: no periodontal therapy TG: non-surgical periodontal therapy (oral hygiene instruction, scaling and root planning)	VPI, GPI, PRF, BOP, PPD and CAL	BP, TG, HDL-C, glucose, HbA1c, CRP and HOMA index
Lobo et al, 2020 Brazil^28^	RCT	CG = 24 (54.6 ± 6.7) TG = 24 (52.7 ± 9.3)	Clinical follow-up at 6 months	Adults with myocardial infraction and severe periodontitis	CG: no periodontal therapy TG: non-surgical periodontal therapy (oral hygiene instruction, scaling and root planning)	PPD, CAL, BOP, VP	FMD, IL-1β, IL-6 and IL-10
Montero et al, 2020 Spain^35^	RCT	CG = 31 (58.3 ± 5.8) TG = 32 (56.7 ± 6.5)	Clinical follow-up at 3- and 6- months	MetS patients with periodontitis stages 3–4	CG: dental prophylaxis (supragingival plaque removal, oral hygiene instruction) TG: non-surgical periodontal therapy (supragingival plaque control, oral hygiene instruction, subgingival scaling and root)	PPD, CAL, BOP, PII, GI	Hs-CRP, α-1 antitrypsin, WBC, HbA1c, FPG, insulin, creatinine, TC, HDL-C, LDL-C, IL-1β, IL-6, IL-18, TNF-α
Seinost et al, 2020 Austria^49^	RCT	CG = 30 (59 ±8.4) TG = 30 (58.7 ± 7.9) + 29 (59.6 ± 8.5)	Clinical follow-up at 3 months	PAD patients with periodontitis	CG: oral hygiene instruction TG: non-surgical periodontal therapy (oral hygiene instruction, scaling and root planning)	BOP, PPD and CAL	Vascular inflammation via PET/CT
Montenegro et al, 2019 Brazil^34^	RCT	CG = 43 (60.8 ± 8.5) TG = 39 (58.4 ± 9.2)	Clinical follow-up at 3 months	Adults, with a diagnosis of stable CAD and severe chronic periodontitis	CG: dental prophylaxis (supragingival plaque removal, oral hygiene instruction) TG: non-surgical periodontal therapy (supragingival plaque control, oral hygiene instruction, subgingival scaling and root planning)	VPI, GBI, GR, PPD, BOP and CAL	CRP Lipid and glycemic profiles (glucose, HbA1c, TG, TC, HDL-C, LDL-C) Systemic cytokines (IL-1β, IL-6, IL-8, IL-10, IFN-γ, and TNF-α)
Saffi et al, 2018 Brazil^43^	RCT	CG: N = 38 (61.7 ± 8.3) TG: N = 31 (58.6 ± 8.5)	Clinical follow-up at 3 months	Adults diagnosed stable CAD and severe chronic periodontitis	CG: dental prophylaxis (supragingival plaque removal, oral hygiene instruction) TG: non-surgical periodontal therapy (supragingival plaque control, oral hygiene instruction, subgingival scaling and root planning)	VPI, GR, PPD, BOP and CAL	FMD mesuring, CRP, lipid and glycemic profiles (glucose, HbA1c, TG, TC, HDL-C, LDL-C)
Zhou et al, 2017 China^65^	RCT	CG: N = 54 (38.38 ± 9.31) TG: N = 53 (41 ± 8.64)	Clinical follow-up at 1-, 3- and 6- months	Prehypertensive adults with moderate to severe periodontitis	CG: dental prophylaxie (supragingival plaque removal, oral hygiene instruction) TG: non-surgical periodontal therapy (supragingival plaque control, oral hygiene instruction, subgingival scaling and root planning) + minocycline hydrochloride ointment	PPD, CAL and BOP	BP, EMPs, CRP and IL-6
Ren et al, 2016 China^41^	RCT	CG: N = 47 (45.0 ± 9.2) TG: N = 46 (44.5 ± 10. 1)	Clinical follow-up at 1 month	Adults with moderate to severe periodontitis	CG: dental prophylaxis (supragingival plaque removal, oral hygiene instruction) TG: non-surgical periodontal therapy (supragingival plaque control, oral hygiene instruction, subgingival scaling and root planning)	PPD, CAL and BOP	baPWV, ABI, NP, CRP and IL-6
Caúla et al, 2014 Brazil^6^	RCT	CG = 32 (44 ± 6.5) TG = 32 (44.4 ± 5.9)	Clinical follow-up at 2- and 6- months	Adult with severe and chronic periodontitis	CG: no periodontal therapy TG: non-surgical periodontal therapy (supragingival plaque control, oral hygiene instruction, subgingival scaling and root planning)	PPD, CAL BOP and plaque index	CRP, erythrocyte sedimentation rate, lipid profile (TG, TC, HDL-C, LDL-C)
Kapellas et al, 2014 Australia^16^	RCT	CG: N = 135 (40.3 ± 9.6) TG: N = 138 (40.2 ± 10.9)	Clinical follow-up at 3- and 12- months	Adults with moderate periodontitis	CG: oral hygiene instruction TG: non-surgical periodontal therapy (oral hygiene instruction, scaling and root planning)	CAL, PPD and VPI	PWV and IMT
Bokhari et al, 2012 Pakistan^3^	RCT	CG: N = 105 (50.1 ± 0.9) TG: N = 212 (49 ± 0.6)	Clinical follow-up at 1- and 2 months	Adult diagnosed CHD case, with periodontitis	CG: no periodontal therapy TG: non-surgical periodontal therapy (supragingival plaque control, oral hygiene instruction, subgingival scaling and root planning)	BOP, PPD and CAL	CRP, fibrinogen, and WBCs
Chen et al, 2012 China^7^	RCT	CG: N = 41 (63.2 ± 8.51) TG 1: N = 42 (59.86 ± 9.48) TG 2: N = 43 (57.91 ± 11.35)	Clinical follow-up at 1,5-, 3- and 6 months	Adults with T2DM and moderate to severe periodontitis	CG: oral hygiene instruction TG 1: non-surgical periodontal therapy (oral hygiene instruction, scaling and root planning) and subgingival debridement at 3 months follow-up TG 2: non-surgical periodontal therapy (oral hygiene instruction, scaling and root planning) and supragingival debridement at 3 months follow-up	PI, BOP, PPD, GR and CAL	HbA1c, hs-CRP, FPG, TC, TG, HDL-C, LDL-C and TNF-α

Li et al, 2011 China^23^	RCT	CG: N = 25 (59.7 ± 10.3) TG: N = 25 (58.6 ± 11.6)	Clinical follow-up at 3 months	Adult with periodontitis	CG: no periodontal therapy TG: non-surgical periodontal therapy (oral hygiene instruction, scaling and root planning)	VPI, PPD and BOP	EPC, PAT index, CRP, Apolipoprotein and lipoprotein
Kamil et al, 2011 Jordan^15^	RCT	CG: N = 18 (45.4 ± 3.3) TG: N = 18 (46.7 ± 3.4)	Clinical follow-up at 3 months	Adult with advanced periodontitis	CG: oral hygiene instruction TG: non-surgical periodontal therapy (oral hygiene instruction, scaling and root planning)	PI, GI and PPD	CRP, lipid profile (TG, TC, HDL-C, LDL-C)
Taylor et al, 2010 Australia^53^	RCT	CG = 64 (55.7 ± 12.2) + 54 (56.1 ± 12.1) TG = 61 (52.1 ± 13.3)	Clinical follow-up at 8 weeks for TG and 12 weeks for CG	Adult patients with periodontitis	CG: no periodontal therapy TG: non-surgical periodontal therapy (oral hygiene instruction, scaling and root planning)	PPD, BOP and LOA	Fibrinogen, CRP, PAI-1, lipid profile (TG, TC, HDL-C, LDL-C) and haematological markers
RCT: randomised control trial, CG: control group, TG: test group, T2DM: type 2 diabetes mellitus, MetS: metabolic syndrome, PAD: peripheral arterial disease, CAD: coronary artery disease, CHD: coronary heart disease, PPD: periodontal probing depth, CAL: clinical attachment level, BOP: bleeding on probing, VPI: visible plaque index, GPI: gingival periodontal index, PRF: presence of plaque retention factors, GI: gingival index, VP: visible plaque, PI/PII: plaque index, GBI: gingival bleeding index, GR: gingival recession, LOA: loss of attachment, IL: interleukin, TNF: tumour necrosis factor, hs-CRP: high-sensitivity C-reactive protein, TG: triglyceride, HDL-C: high-density lipoprotein cholesterol, LDL-C: low-density lipoprotein cholesterol, TC: total cholesterol, FPG: fasting plasma glucose, HOMA: homeostasis model assessment, WBC: white blood cell, NP: neopterin, BP: blood pressure, FMD: flow-mediated dilatation, baPWV: brachial-ankle pulse wave velocity, IMT: intima-media thickness, PAT: peripheral arterial tone, EPC: endothelial progenitor cell, PAI: plasminogen activator inhibitor, PET/CT: positron emission tomography/computed tomography.

The number of subjects included ranged from 18 to 135 in the control groups, whereas the test groups included between 18 and 212 patients. All participants in the test groups received periodontal therapy (supragingival plaque control, oral hygiene instructions, subgingival scaling, and root planning), whereas subjects in the control groups either did not receive any periodontal therapy or received only limited dental prophylaxis (supragingival plaque removal, oral hygiene instructions). Clinical follow-up ranged from 1 to 12 months, with an average follow-up period of 3–6 months.

Some of the trials involved an otherwise healthy population (Ren et al, 2016^41^, Caúla et al, 2014^6^, Kapellas et al, 2014^16^, Li et al, 2011^23^, Kamil et al, 2011^15^, and Taylor et al, 2010^53^). Other trials focused on populations with comorbidities such as metabolic syndrome (MetS) patients (Milanesi et al, 2022^33^ and Montero et al, 2020^35^), peripheral arterial disease (PAD) patients (Seinost et al, 2020^49^), coronary artery disease (CAD) patients (Montenegro et al, 2019^34^ and Saffi et al, 2018^43^), prehypertensive patients (Zhou et al, 2017^65^), patients with myocardial infarction (Lobo et al, 2020^28^), coronary heart disease (CHD) patients (Bokhari et al, 2012^3^), and type 2 diabetes mellitus (T2DM) patients (Kolte et al, 2023^19^ and Chen et al, 2012^7^).

### Study Outcomes

The reported outcome parameters of all trials can be divided into three groups: endothelial function; systemic inflammation and thrombosis; and lipid and glucose metabolism. The main results are described in Table 3.

**Table 3:** Main findings of the included studies

		Study	Main finding
Vascular function	Otherwise healthy	Ren et al, 2016^41^	Significant difference in baPWV reduction in TG after 1 month (P <0.001). baPWV was significantly lower in TG than CG (P <0.05). No significant difference in the change of ABI between CG and TG. Significant correlation between the change of clinical periodontal index and change of baPWV (P <0.001), the decrease of circulin baPWV was positively correlated with the reduction in neopterin (P <0.001) in TG.
Kapellas et al, 2014^16^	No significant difference in PWV values in TG. Significant difference in maximum carotid IMT reduction in TG than in CG at 12 months (P = 0.031).
Li et al, 2011^23^	Significant difference circulating CD34+ cell count reduction in TG (P = 0.011). No significant differences in other subsets of CPCs counts between CG and TG. The reduction of CD34+ cell count was positively correlated with the decrease in site % with BOP (P = 0.005), site % with PD > 4 mm (P <0.001), apolipoprotein A1 (P = 0.015) and lymphocytes (P = 0.008). Neutral effect on peripheral vascular endothelial function (via PAT index) after NSPT.
With comobidity	Milanesi et al, 2022^33^	No significant difference in regard to blood pressure at the 3- and 6-month visits between CG and TG.
Seinost et al, 2020^49^	No significant difference in vascular inflammation reduction in either the carotid and lower leg arteries and in the aorta.
Lobo et al, 2020^28^	FMD significantly improved in TG (P = 0.01) than in CG, significant difference between CG and TG (P = 0.03).
Montero et al, 2020^35^	Significant difference in SBP reduction values at 3 months (P = 0.008) and in DBP values reduction at 3 and 6 months (P = 0.0019 and P = 0.009) in TG.
Saffi et al, 2018^43^	No significant improvements in FMD after 3 months in TG and between CG/TG.
Zhou et al, 2017^65^	Significant difference in SBP values reduction at 1, 3 and 6 months (P <0.05) and in DBP values reduction at 3 and 6 months (P<0.01) in TG. Significant difference in EMPs reduction at 3 and 6 months (P<0.01) in TG. Significant correlation between the changes (reduction) in SBP (P = 0.009), DBP (P = 0.008) and EMPs (P <0.001) at 6 months after PT.
Markers of systemic inflammation and thrombosis	Otherwise healthy	Ren et al, 2016^41^	Significant difference in NP reduction (P <0.001), hs-CRP (P <0.001), and IL-6 (P <0.001) in TG at 1 month.
Caúla et al, 2014^6^	Significant difference in serum CRP levels reduction only at 6 months in TG (P <0.001) and between CG/TG at 2 months (P = 0.001) and at 6 months (P <0.001). Serum CRP levels increased significantly at 2 months (P = 0.003) and at 6 months (P < 0.001) in CG.
Taylor et al, 2010^53^	Significant difference in fibrinogen reduction in TG at 8 weeks. No significant difference in concentration of CRP, PAI-1 and vWF.
Kamil et al, 2011^15^	Significant difference in concentration of serum CRP reduction in TG (P <0.005). There is a significantly correlation between the reduction of CRP and the reduction in PI, GI and PPD.
With comobidity	Kolte et al, 2023^19^	Significant difference in increase IL-10 (P <0.0001) and in reduce TNF-α (P <0.0001) and hs-CRP (P <0.0001) in TG than in CG. There is also a significant difference between groups (P <0.0001) in all parameters.
Milanesi et al, 2022^33^	No significant difference in CRP at 3- and 6- months between CG and TG.
Lobo et al, 2020^28^	No statistically significant differences between baseline and 6-month values of interleukin-1B, interleukin-6 and interleukin 10 in either group.
Montero et al, 2020^35^	Significant difference in serum CRP levels reduction at 3- and 6- months between CG and TG (P = 0.001 and P = 0.004). Significant difference in IL-1β (P = 0.046) and TNF-α (P = 0.037) reduction between CG and TG at 3 months only. Significant difference in TG compared to baseline in CRP (3–6 months), IL-1β (3 months), IL-8 (3 months), TNF-α (3–6 months).
Montenegro et al, 2019^34^	Significant difference in the increase in serum CRP levels (patients with CRP<3mg/L at baseline) in CG (P = 0.01), compared with TG where CRP remained unchanged at 3 months. There is therefore a significant difference between CG and TG. For patients with CRP >3mg/L at baseline, there is a significant difference between CG and TG (P = 0.01) at 3 months. Significant difference in serum CRP levels reduction (patients with CRP ≥ 3 mg/L at baseline) in TG (P = 0.04), but no significant difference in CG. Significant difference in concentration IL-6 (P = 0.04) and IL-8 (P = 0.04) reduction in TG at 3 months.
Saffi et al, 2018^43^	Significant difference in increase in sVCAM-1 (P = 0.03) and sICAM-1 (P = 0.03) in CG at 3 months but no significant difference in TG, resulting a significant difference between CG and TG at 3 months (P = 0.04 and P = 0.01). No significant difference in P-selectin at 3 months.
Zhou et al, 2017^65^	Significant difference in hs-CRP reduction (P <0.001) at 3- and 6- months in TG and in IL-6 (P = 0.018) only at 6 months in TG.
Bokhari et al, 2012^3^	Significant difference in serum CRP levels reduction at 1- and 2 months in TG (P <0.001) and between CG and TG at 1 month (P = 0.034) and 2 months (P = 0.007). Significant difference in serum fibrinogen levels reduction in TG and between CG and TG only at 2 months (P = 0.010).
Chen et al, 2012^7^	Significant difference in serum hs-CRP levels reduction at 1.5- 3- and 6 months follow-up in TG 1/2 (P <0.05). No significant difference in TNF-α between TG 1/2 and CG at any follow-up.

Lipid and glucose metabolism	Otherwise healthy	Caúla et al, 2014^6^	Significant difference in TC levels reduction in TG (P <0.001) and between CG and TG (P = 0.021) at 6 months. Significant difference in TG reduction at 6 months only (P = 0.015).
Kamil et al, 2011^15^	No significant difference in concentration in TC, HDL-C, LDL-C and TG at 3 months follow-up in both groups.
Taylor et al, 2010^53^	Significant difference in the mean total cholesterol reduction in CG at 12 weeks. HDL increases significantly in TG at 8 weeks.
With comobidity	Kolte et al, 2023^19^	The mean fasting blood glucose level was statistically significant in TG (P = 0.0001), and in CG (P = 0.008) at 6 months follow-up and between CG and TG (P <0.0001). The post-meal blood glucose levels are statistically significant in TG at 6 months (P <0.0001)and also between CG and TG (P <0.0001). HbAIc significantly decreased in TG at 6 months (P <0.0001) and between CG and TG (P <0.0001).
Montero et al, 2020^35^	Significant difference in HbA1c reduction in TG compared to baseline (3 and 6 months) and between CG and TG at 3 months onlay (P = 0.013). No difference in fasting plasma glucose, TC, HDL-C and LDL-C.
Chen et al, 2012^7^	HbAIc significantly decreased only in TG2 at 6 months (P <0.05). FPG significantly decreased only in TG1 at 6 months (P <0.05). No significant difference in HbA1c, FPG, lipid metabolic levels between TG 1/2 and CG at any follow-up.
CG: control group, TG: test group, baPWV: brachial-ankle pulse wave velocity, ABI: ankle-brachial index, IMT: intima-media thickness, CD: cluster differentiation, CPC: circulating progenitor cell, PAT: peripheral arterial tone, FMD: flow-mediated dilatation, SBP: systolic blood pressure, DBP: diastolic blood pressure, EMP: endothelial micro-particule, NP: Neopterin, hs-CRP: high-sensitivity C-reactive protein, IL: interleukin, PAI: plasminogen activator inhibitor, vWF: Von Willebrand factor, TNF: tumour necrosis factor, sVCAM: soluble vascular cell adhesion molecule, sICAM: soluble intercellular adhesion molecule, TC: total cholesterol, TG: triglyceride, HDL-C: high-density lipoprotein cholesterol, LDL-C: low-density lipoprotein cholesterol, FPG: fasting plasma glucose.

#### Non-surgical periodontal therapy and endothelial function

Endothelial function has been assessed via various methods. Studies have investigated blood pressure, arterial diameter/stiffness via pulse wave velocity (PWD), the ankle-brachial index (ABI), flow-mediated dilation (FMD), and vascular inflammation via PET/SCAN analysis. Two studies measured endothelial function by examining endothelial microparticles (CD34+, CD31+, CD42-…). Endothelial function tended to improve, and in particular, the concentration of endothelial microparticles was reduced in patients who had undergone periodontal therapy.

##### Non-surgical periodontal therapy and systemic inflammation and thrombosis

The majority of studies did not show a significant decrease in C-reactive protein (CRP), interleukin (IL-6), IL-8, IL-10, IL-1β, TNF-α and fibrinogen levels after NSPT, both in the healthy population and in patients with comorbidities. Other inflammatory and thrombotic biomarkers, including sVCAM-1/sICAM-1 and neopterin, which significantly decreased after NSPT, were studied. However, the concentrations of PAI-1 and von Willebrand factor (vWF) did not change after NSPT.

Lipid metabolism was assessed by measuring the concentrations of total cholesterol, HDL-L, LDL-C and triglycerides. After NSPT, the values remained unchanged in the majority of the studies. However, two studies reported a significant reduction in TC in the TG. Glucose metabolism was assessed by measuring the HbAIc concentration, fasting blood glucose level and post-meal blood glucose level. There was a significant difference in the reduction in these markers in patients who underwent NSPT.

### Analysis of the Risk of Bias

**Fig 2:**
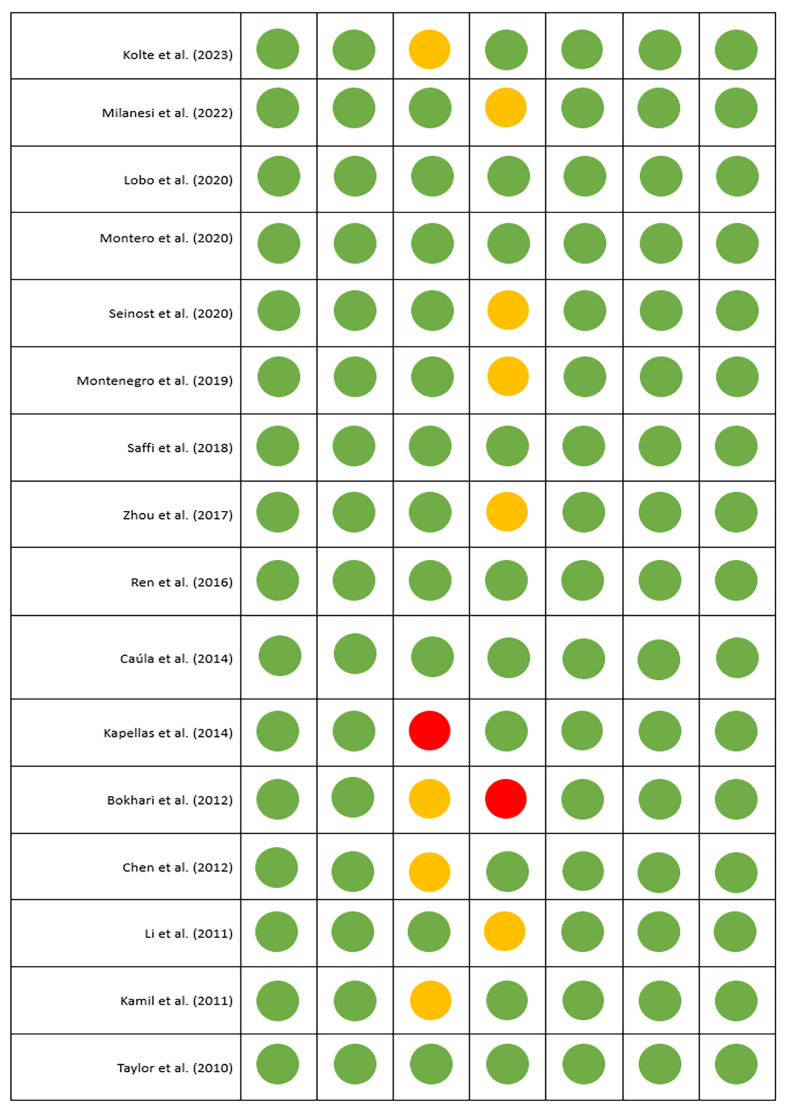

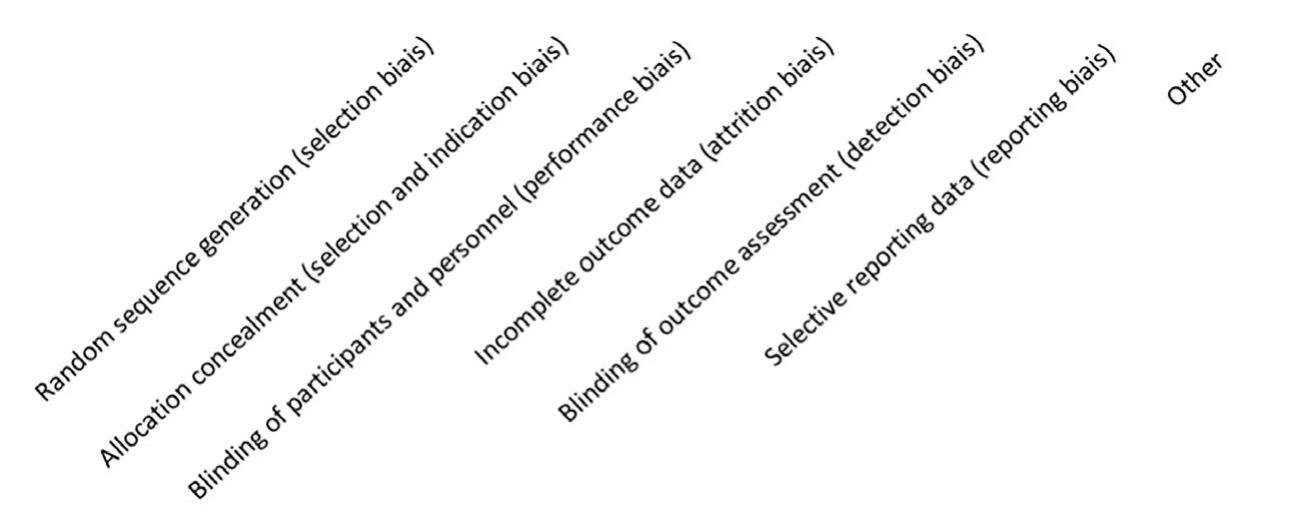
Risk of bias of included studies. Green indicates low risk of bias, orange indicates uncertain or moderate risk of bias, and red indicates high risk of bias.

The analysis of the risk of bias, as described in Figure 2, revealed a low-to-moderate level of risk for all items across the different studies. The risk of selection, indication, and reporting bias is generally low due to the randomised nature of the included studies. The risk of attrition bias is mostly low or moderate, except in the study by Bokhari et al,^3^ which had a high level of bias because only 80% of participants completed the study.

Notably, the studies by Kolte et al,^19^ Bokhari et al,^3^ Chen et al,^7^ and Kamil et al^15^ have a moderate level of risk of bias, whereas the study by Kapellas et al^16^ has a high risk of bias for the item ‘blinding of participants and personnel’. This performance bias could be explained by the lack of blinding of personnel or failure to report blinding. The comparability of the data between the test and control groups was very good, and no bias was detected.

## DISCUSSION

Numerous meta-analyses and systematic reviews have confirmed the close link between periodontal diseases and cardiovascular diseases.^10,17,60,63^ A recent French cohort study examined patients with acute myocardial infarction and various outcomes, including periodontitis. This issue is therefore highly relevant.^12^

Our systematic review focused on the effects of non-surgical periodontal therapy on biomarkers of cardiovascular risk. This systematic review included 16 RCTs published between 2010 and 2024 to ensure a sufficient level of evidence and to strengthen the methodological robustness of this review. Six of these studies involved a healthy population, while the other ten included participants with cardiovascular or systemic comorbidities. The choice of including healthy patients or those with comorbidities enabled the results to be assessed on a population panel that was more representative of the general population. In addition, the review includes an extended analysis of several biomarkers linked to cardiovascular risk (endothelial function, inflammation, thrombosis, lipid and glucose metabolism), providing a comprehensive overview.

Initially, we investigated the effect of non-surgical periodontal treatment on endothelial function. The available studies allow us to conclude that the effect of periodontal treatment on endothelial function is positive but remains uncertain. To measure vascular function, we relied on assessments of arterial pressure, arterial stiffness (using various methods), vascular inflammation, and the concentration of endothelial microparticles.

With respect to the effects of NSPT on blood pressure and arterial stiffness, we observed a positive trend in the improvement of these parameters, which is in line with the scientific literature. However, this positive trend remains uncertain.^29,36,50^ Two RCTs reported significant improvements in systolic blood pressure (SBP) and diastolic blood pressure (DBP) values after NSPT. The data in scientific literature remains limited on this point and does not provide definitive conclusions. The improvement in arterial stiffness after NSPT is better documented in the literature.^8,48^ Two meta-analyses have demonstrated an association between periodontal disease and increased arterial stiffness. However, the effect of periodontal therapy remains debatable. In this systematic review, only three RCTs reported improvements in endothelial function based on arterial stiffness measurements (via flow-mediated dilatation, brachial-ankle pulse wave velocity, and carotid intima-media thickness).

One RCT examined vascular inflammation via PET/CT analysis. Although the conclusions did not show any improvement after periodontal therapy, the procedure appears to be promising. More studies are needed to compare results effectively.

Over the past ten years, various studies have focused on the effects of periodontal treatment on the concentrations of endothelial microparticles. In 2009, Li et al demonstrated for the first time that moderate to severe chronic periodontitis was associated with an increased level of circulating endothelial progenitor cells (EPCs).^22^ In this systematic review, two RCTs demonstrated a significant decrease in the number of EPCs, notably CD31+, CD42–, and CD34+, three or six months after NSPT. Further studies are necessary to confirm these findings. We also investigated the effects of NSPT on inflammation and thrombosis biomarkers. The main finding of the present study was that CRP was significantly reduced after NSPT. This improvement in CRP values was observed both in healthy individuals and those with comorbidities. CRP is a predictive marker of inflammation. A recent meta-analysis confirmed that periodontal treatment reduced CRP levels.^30,46^

Proinflammatory cytokines also have detrimental effects on cardiovascular risk. Several proinflammatory immune mediators are expressed and released into the circulation in response to local inflammation, affecting other organ systems. Inflammation in the periodontium has systemic repercussions.^26,31,64^ In this study, four RCTs demonstrated a significant decrease in the concentration of proinflammatory cytokines following periodontal therapy.

One RCT examined cell adhesion molecules, which are biomarkers of inflammation. Saffi et al^43^ reported an increase in these molecules in the control group but did not demonstrate a significant decrease in the periodontal therapy group. Few studies are available on cell adhesion molecules and their relationship with periodontal diseases.^2,51,56^ Further investigations in this area would be beneficial.

According to the literature, there is a close link between periodontal diseases and fibrinogen levels.^20,57^ However, a recent study was unable to establish a connection between periodontal treatment and a decrease in fibrinogen levels.^1^ In this systematic review, two RCTs reported a significant decrease in fibrinogen levels after NSPT.

In our final section, we examined the effects of NSPT on lipid and glucose metabolism. In our systematic review, lipid levels (triglycerides, total cholesterol, and HDL-C) improved in patients with periodontal diseases compared with those in the control group. However, these results need confirmation through additional studies. In our present study, the RCTs were not unanimous in terms of the improvement in the lipid profile after NSPT. Nevertheless, these results are consistent with the literature. A meta-analysis by Lianhui et al (2017)^24^ highlighted a correlation between periodontitis and hyperlipidaemia; meanwhile, the meta-analysis by Garde et al (2019)^11^ suggested the potential benefits of periodontal treatment in reducing total cholesterol and triglyceride levels.^11,24^

The effect of periodontal therapy on glucose metabolism was evaluated in three RCTs involving patients with comorbidities (two RCTs involving patients with type 2 diabetes mellitus (T2DM) and one RCT involving patients with metabolic syndrome (Mets). The results revealed a significant reduction in glycated haemoglobin (HbA1c) and fasting plasma glucose (FPG) after three or six months of follow-up. The scientific literature has shown for many years that periodontal treatment improves glucose metabolism, particularly HbA1c and FPG, in patients with T2DM.^9,21^

This review has several limitations. Firstly, there is considerable heterogeneity between the studies included, particularly in terms of the populations studied, treatment protocols, follow-up times and measurement methods. These disparities complicate a uniform analysis and restrict the generalisability of the results. Although the review covers a diverse population, the small size and limited geographical diversity of the studies analysed may hamper the representativeness of the conclusions. Furthermore, the relatively short duration of follow-up in most of the studies does not allow the long-term effects of periodontal treatment to be assessed. Finally, although the review establishes correlations between periodontal treatment and cardiovascular biomarkers, it does not investigate the underlying mechanisms involved. This review has several strong points. It addresses a relevant and topical subject, which is particularly important given the high prevalence of periodontal and cardiovascular disease. The inclusion of recent studies with a high level of evidence (RCT) guarantees reliable results in line with current scientific advances. In addition, the review explores a wide range of cardiovascular biomarkers, providing a comprehensive and detailed view of the potential effects of periodontal treatment on cardiovascular health.

NSPT shows moderate to high clinical relevance by improving several markers of vascular function (PWV, FMD, IMT, blood pressure) and systemic inflammation (CRP, IL-6, TNF-α) in both healthy and comorbid patients, with a notable benefit on glycaemic control (HbA1c, FPG), particularly in individuals with metabolic risk, although its effects on lipid profile remain limited and heterogeneous.

## CONCLUSION

In conclusion, this systematic review highlights the growing evidence of the link between non-surgical periodontal therapy (NSPT) and improved biomarkers of cardiovascular risk. By including 16 RCTs from diverse populations and exploring a wide range of biomarkers, the review provides a comprehensive perspective on the systemic effects of periodontal therapy. Positive trends were observed in endothelial function, arterial stiffness, inflammation, lipid profiles and glucose metabolism, suggesting that periodontal treatment may play a significant role in cardiovascular risk management. In particular, the significant reductions in CRP and proinflammatory cytokines, as well as improvements in certain lipid and glycaemic parameters, reinforce the strong interconnection between oral and cardiovascular health.

However, these results are tempered by major limitations, notably the heterogeneity of the populations studied, treatment protocols, follow-up times and measurement methods. These discrepancies prevent the results from being generalised, and underline the need for robust, standardised clinical trials with long-term follow-up. Furthermore, although correlations between periodontal therapy and cardiovascular biomarkers are apparent, the underlying mechanisms remain insufficiently explored.

Despite these challenges, this review highlights the potential of periodontal therapy to offer benefits beyond oral health, particularly for those at high cardiovascular risk. Future research should focus on filling the gaps identified, refining our understanding of the mechanisms involved and validating these findings. An interdisciplinary approach involving oral and cardiovascular health professionals is essential to optimise the prevention and management of cardiovascular disease through improved periodontal care.
